# The impact of Anastrazole and Letrozole on the metabolic profile in an experimental animal model

**DOI:** 10.1038/srep17493

**Published:** 2015-12-01

**Authors:** Ioannis Boutas, Vasilios Pergialiotis, Nicolaos Salakos, George Agrogiannis, Panagiotis Konstantopoulos, Laskarina-Maria Korou, Theodoros Kalampokas, Odysseas Gregoriou, George Creatsas, Despina Perrea

**Affiliations:** 1 Second Department of Obstetrics and Gynecology, University of Athens, Vas. Sofias 76, Athens, 11528, Greece; 2 Laboratory of Experimental Surgery and Surgical Research “N.S. Christeas”, Athens Medical School, Agiou Thoma 15B, Athens, 11527, Greece; 3 First Department of Pathology, School of Medicine, National University of Athens, Mikras Asias 75str, Athens, 11527, Greece.

## Abstract

Anastrazole and Letrozole are used as endocrine therapy for breast cancer patients. Previous studies suggested a possible association with metabolic and liver adverse effects. Their results are conflicting. Fifty-five 4-week-old female Wistar rats were allocated in 4 groups 1) ovariectomy control (OC), 2) ovariectomy-Anastrazole (OA) 3) ovariectomy -Letrozole (OL), 4) control. Serum glucose, cholesterol, triglycerides, HDL-c and LDL-c were measured at baseline, 2 and 4 months. At the end, the animals‘ liver were dissected for pathology. At 4 months, total cholesterol differed among the OC and OL groups (*p *=* 0.15*) and the control and OL groups (*p *=* 0.12*). LDL-C differed between the control and OC groups (*p *=* 0.*015) as well as between the control and OA (*p *=0* .*015) and OL groups (*p *=* 0.002*). OC group triglycerides, differed from those of the OL group (*p *=0* .002*) and the control group (*p *=* 0.007*). The OA also significantly differed from the OL (*p *=* 0.50*). Liver pathology analysis revealed differences among groups with favored mild steatosis and ballooning. Anastrazole and Letrozole seem to negatively influence the lipid profile in our experimental model. This information should be taken in caution by medical oncologists when addressing patients with altered lipid metabolism.

Aromatase is the main enzyme which catalyzes the conversion of androgen to estrogen in the adipose tissue of postmenopausal women^1^. It is present in numerous tissues including the ovaries, placenta, skin, adipose tissue and breast cells^2^. Aromatase inhibitors (AIs) exhibit anti-estrogenic activity which is triggered by inhibiting the cytochrome P450^3^. Three generations of AIs have been developed^4^. The third generation AIs present diversities concerning their effects on the lipid profile of women who suffer from breast cancer^5^. Despite the fact that they seem to demonstrate improved tolerability^6^, studies on Anastrazole and Letrozole indicate a possible negative impact on liver function of postmenopausal women^7^,^8^,^9^. However, data seem to be conflicting in this field. Specifically, based on the final results of the National Surgical Adjuvant Study-BC 04, Anastrazole did not influence serum lipids^10^. On the other hand, in the ATAC trial, hypercholesterolemia was more prevalent among women treated with Anastrazole^11^. Letrozole treatment was correlated with increased serum cholesterol levels in the BIG 1–98 trial^12^. It has been also shown that low levels of estrogens affect liver metabolism in mice in numerous ways, such as lipid accumulation and hepatic steatosis^13^,^14^.

The aim of the present study is to investigate whether Anastrazole and Letrozole when administered in ovariectomized female rats influence their lipid profile and the liver architecture.

## Results

At enrollment, the mean body weight of animals did not significantly differ among groups (Table 1). Similarly, baseline serum glucose, cholesterol, triglycerides and LDL-c levels were also comparable (Table 2).

Two months after the initiation of the experiment the total cholesterol levels significantly differed among groups (*p *=* 0.003*). At post-hoc analysis this resulted from differences detected between the ovariectomized control and the Letrozole groups (*p *=* 0.001*) as well as between the Anastrazole and the Letrozole groups (*p *=* 0.03*). In line with these observations, serum HDL-C levels significantly differed between the ovariectomized control and the Letrozole groups (*p *=* 0.001*) and between the Anastrazole and the Letrozole groups (*p *=* 0.025*). Serum triglycerides concentration was also differently affected and at post-hoc analysis differences were evident only between the ovariectomized control and Anastrazole groups (*p *=* 0.01*) and the Anastrazole and Letrozole groups (*p *=* 0.3*).

**Figure 1:**
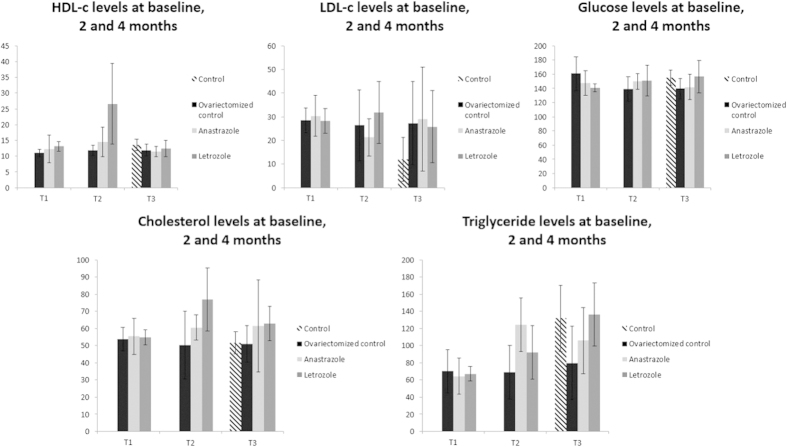
Serum glucose (mg/dl), total cholesterol (mg/dl), LDL-cholesterol (mg/dl), HDL-cholesterol (mg/dl) and triglycerides levels (mg/dl) at baseline (T1), at two months (T2) and at the end of the experimental period (T3). The data are presented as Mean ± Standard Deviation (Groups: Control; Ovariectomized control; Anastrazole; Letrozole)

At the end of the study, mean body weight levels remained comparable among the different groups (Table 1). Regarding serum lipid levels, the majority of the aforementioned differences persisted. Specifically post-hoc analysis for total cholesterol revealed significant differences only among the ovariectomized control and the Letrozole groups (*p *=* 0.15*) and the control and Letrozole groups (*p *=* 0.12*). LDL-C was also affected and the statistical significance was evident among the ovariectomized control group and the control group (*p *=* 0.15*) as well as among the Anastrazole and control groups (*p *=* 0.15*) and Letrozole and control groups (*p *=* 0.19*). In the case of triglycerides levels, the ovariectomized control group differed from both the Letrozole group (*p *=* 0.2*) and the control group (*p *=* 0.07*). The Anastrazole group also significantly differed from the Letrozole group (*p *=* 0.5*) (Fig. 1).

**Figure 2:**
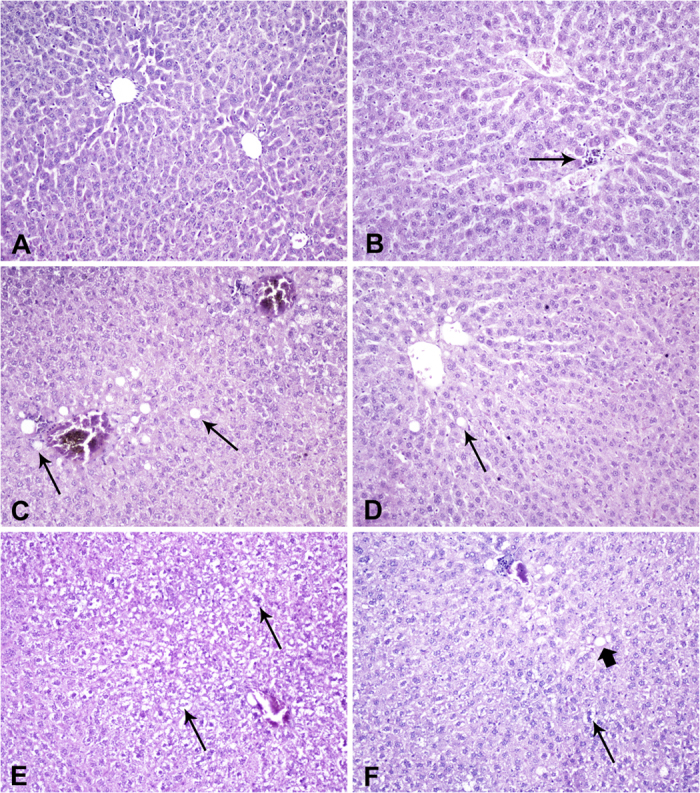
Representative liver figures, eosin-hematoxylin staining, 200× original magnification. (**A**) normal liver architecture. (**B**) small focus of periportal chronic inflammatory infiltrate (arrow). (**C**) moderate—score 2—parenchymal steatosis with panacinar distribution (arrows). (**D**) mild—score 1—steatosis (arrow). (**E**) moderate number of ballooned hepatocytes—score 2—(arrows). (**F**) focal hepatocyte ballooning—score 1—(arrow) along with sparse lipid droplets (thick arrow).

Hematoxylin-eosin stained liver samples obtained from animals of Letrozole and Anastrazole groups showed signs of hepatic steatosis and ballooning (Fig. 2, Table 3). The grade of fatty liver disease was considered as “mild” in eight of the eleven rats in Anastrazole group. In nine of the twelve rats of the Letrozole group, the grade of steatosis was considered as “mild”, in two animals of this group the grade of steatosis was characterized as “moderate” while only one animal of this group presented normal liver architecture. In both control and ovariectomized control groups, “mild” steatosis was detected in one animal per group. No statistically significant differences were detected in the grade of steatosis between the Letrozole and Anastrazole groups (*p *=* 0.331*) although liver architecture was more disturbed in Letrozole treated rats. Hepatocellular degeneration (ballooning) of grade 1 was confirmed in five of the twelve animals of Letrozole group and in two of the eleven animals of Anastrazole group. Ballooning of grade 2 was detected in two Letrozole treated rats. Ballooning was not observed in any animal of the control or ovariectomized control groups. Neither portal nor lobular inflammation were detected in the liver lesions of all the animals studied.

## Discussion

Third generation AIs are largely used in postmenopausal women with a diagnosis of hormone receptor positive breast cancer^15^. Naturally, their safety and effectiveness are improved compared to the earlier generation AIs^16^. The menopausal transition and the postmenopausal period influence the cardiovascular system directly and indirectly. Several studies have demonstrated the crucial role of total cholesterol, LDL–C and triglycerides as important risk factors for cardiovascular events^17^,^18^,^19^. Female sex hormones have been correlated to a decline in the incidence of cardiovascular events in young and middle-aged women as compared to men, while adverse changes in serum total cholesterol and triglyceride levels between pre and postmenonopausal period have been reported^20^. Thus, estrogen influence positively serum cholesterol levels and AIs can interrupt this interplay thus increasing the odds of a developing cardiovascular disease^21^.

The majority of the studies concerning the effects of Anastrazole on lipid profile have shown an increase in HDL-C levels and various effects on LDL-C and triglyceride levels^22^,^23^. In a large systematic review and meta-analysis performed by Amir *et al*., prolonged use of AIs was associated with significant changes of the lipid profile, including hypercholesterolemia^24^. In the same study, the use of AIs was also associated with a higher risk of cardiovascular disease. In the BIG 1–98 trial this risk was documented when studying the effects of administration of Letrozole as compared with tamoxifen^25^. Counterintuitively however, the MA-17 trial showed no changes in terms of lipid profile with Letrozole use^26^.

Several studies have investigated the influence of Anastrazole on the lipid profile of women reporting conflicting results. The ATAC trial suggested that Anastrazole did not affect the lipid profile or the odds of developing cardiovascular disease^27^. The SABRE trial, also reported no differences following Anastrazole administration on LDL-C, HDL-C, or triglyceride levels for a 12-months treatment period^28^. Lin et al observed that treatment with Anastrazole seems to result in less lipid accumulation in hepatic tissue as compared to tamoxifen and concluded that it may be preferable for patients with potential hepatic dysfunction^29^. Furthermore, Sawada et al suggested that Anastrazole may also exert a beneficial effect on the lipid profile of postmenopausal women^30^. Conversely however, the results of the ITA trial, pointed towards lipid metabolism disorders^31^.

Contrary to Anastrazole, the effect of Letrozole on lipid profile and hepatic architecture has been seldom investigated. In a small study which recruited 20 postmenopausal women, Letrozole was associated with a significant increase in total and LDL cholesterol levels 16 weeks after the initial enrollment of the patients^32^. However, these results were not confirmed by the NCIC CTG MA.17 study^33^,^34^.

It has been shown that the occurrence of metabolic syndrome is increased among women after menopause^35^. Modifications on lipid metabolism or inflammatory mediated processes are involved in the action of estrogen deficiency on hepatic function and histology^36^,^37^. The increase in accumulation of fat in the hepatic tissue recorded in our study in the animal groups treated with Anastrazole and Letrozole may be attributed to the inhibition of estrogen production caused by these agents and the subsequent disturbed lipid accumulation.

The effect of Anastrazole and Letrozole on liver function have not yet been clarified. In an experimental study, which was conducted in order to determine the effects of Letrozole on hepatic function in female rats, hepatotoxicity was observed, while minimal histological findings were detected^8^. In a recent study by Lin Y *et al*., Anastrazole was demonstrated to have minimal toxicity in terms of liver function compared to that of tamoxifen^29^. According to the conclusions of a case report, a potential autoimmune mechanism of hepatotoxicity has been also documented in a patient receiving Anastrazole^7^.

A limitation of the study was the induction of the control rats that were not subjected to ovariectomy at the end of the experimental period that did not allow the investigation of potential differences in serum lipid levels among estrogen deficient or not rats throughout the entire protocol.

The results of our study suggest that Letrozole significantly alters the lipid profile of ovariectomized mice, therefore, putting into question its tolerability which is reported by previous clinical studies. Anastrazole on the other hand seems to exert a mild effect on the levels of LDL-c which is not reflected in the total cholesterol and triglyceride levels. Mild histological liver alterations seem also to occur and these alterations should be taken in mind in future clinical studies. Once again Letrozole resulted more cases of mild and moderate liver pathology, although this result did not reach statistical significance.

*Implications for clinical practice and future research.* 

Letrozole’s mode of action on the lipid profile of patients should be seriously evaluated by medical oncologists when addressing patients with altered lipid metabolism until further evidence become available. Anastrazole on the other hand seems to exhibit a milder effect. Future trials should thoroughly investigate the potential metabolic and liver adverse effects of Anastrazole and Letrozole and consistently observe the enrolled patients over an adequate period of time (preferably until the end of the treatment).

Concluding, according to the findings of our study, Letrozole administration over a 2 and 4 month treatment period negatively affects serum lipid metabolism in ovariectomized female rats and disturbs liver histopathology. Anastrazole, on the other hand, seems to result mild changes and might be a safer alternative for ovariectomized patients. Future clinical trials are needed to corroborate our findings because current clinical evidence in the field are scarce and not sufficient to support the tolerability of these drugs.

## Materials and Methods

### Animals

Fifty-five 4-week-old female Wistar rats (Hellenic Pasteur Institute, Department of Animal Models for Biomedical Research, Greece) were maintained in weather controlled chambers (temperature 20 ± 1 °C, humidity 55 ± 5%) under controlled lightning (12 hours light per day) for 30 days in order to adapt to their new environment. ELVIZ 510 food pellets were provided ad libitum, in order to ensure a full nutrient diet. The protocol was approved by the Ethics Committee of the Athens Medical School and by the Veterinary Directorate of Attica Region in agreement with the Directive 2010/63/EU. The methods were carried out in “accordance” with the approved guidelines. The night before the operation food was deprived from the animals.

### Surgical procedures

Forty-five female Wistar rats underwent surgical ovariectomy. The surgical procedures were performed between 8:00 am and 9:00 am on diestrous day 1 (D-1). The animals were anesthetized with a combination of ketamine (75 mg/kg) and xylazine (10 mg/kg) which were administered intraperitoneally. A midline dorsal skin incision was then performed. The ovarian vessels were clamped and both ovaries were excised. Muscles and skin were sutured to close the incision.

### Animal treatment

After the ovariectomy, the operated animals were randomized in three groups. The first group did not receive any drug regimen (ovariectomized control group). The second group received Anastrazole and the third group received Letrozole. Administration of these regimens was performed according to previous reports^38^. Specifically, Anastrazole was administered p.o. in drinking water, after being dissolved in DMSO solution, in a concentration tested to result in a daily uptake of approximately 0.1 mg/kg of body weight and Letrozole was similarly administered in a concentration tested to result in a daily uptake of approximately 2 mg/kg of body weight. Both agents were administered for a 4-month period.

Blood samples were collected using capillary tubes from the medial retro-orbital venous plexus under light ether anesthesia, at the beginning of the experiment (T1), at 2 months (T2) and at the end of the study (4 months-3) at 9:00 AM after a 12-hour fasting period.

Four months after the initiation of the study, the animals were euthanized. At this point ten control animals of similar age were included in the study as a control group without ovariectomy, in order to observe the potential differences of the three groups as opposed to normal values.

### Enzyme-linked immunosorbent assay (ELISA)

Blood specimens were collected in Vacutainer tubes (BD Diagnostics, NJ, USA). The serum was separated after centrifugation of blood at 3000 rpm for 10 minutes. The specimens were stored at -30 °C until the assay which was performed within two months. Serum concentrations of total cholesterol and of triglycerides were determined using the enzymatic PAP commercial kit (“biosis”—Biotechnological Applications, Athens, GR) and HDL-cholesterol was determined with a cholesterol enzymatic photometric method. LDL-cholesterol was determined by the mathematic model “LDL-cholesterol = Total Cholesterol- (HDL-cholesterol + Triglycerides/5)”.

### Pathology

At the end of the 16-week period, animals were euthanized under ether anaesthesia. Liver were dissected immediately for further histopathological analysis as previously described^39^. Liver sections were stained with hematoxylin-eosin and examined blindly by two independent pathologists under light microscopy. The histologic evaluation was conducted in accordance to the guidelines Pathology Committee of Non-Alcoholic Steatohepatitis Clinical Research Network^40^. The histological features were grouped into 4 broad categories: steatosis, ballooning, portal inflammation and lobular activity. A score from 0 (absence) to 3 (severe) was assigned to each parameter.

### Statistical analysis

The normality of the distributions was assessed with Kolmogorov-Smirnov’s test and graphical methods. All data are expressed as median [range]. We used the Kruskal-Wallis non-parametric test for multiple group comparisons and the Dunn’s test of multiple comparisons for *post-hoc* multiple testing. Comparisons between multiple time points were performed using Friedman’s test with Wilcoxon’s Signed Ranks test for *post-hoc* comparisons. The Chi-square and Fishers exact test were used for analysis of dichotomous variables. Differences were considered as statistically significant if the null hypothesis could be rejected with > 95% confidence (p < 0.05).

## Additional Information

**How to cite this article**: Boutas, I. *et al.* The impact of Anastrazole and Letrozole on the metabolic profile in an experimental animal model. *Sci. Rep.* **5**, 17493; doi: 10.1038/srep17493 (2015).

## Figures and Tables

**Table 1:** Animal body weight levels (g) at baseline (1), 2 months (2) and four months (3).

*Variable*	*Ovariectomy*	*Anastrazole*	*Letrozole*	*Control*
Weight				
* Weight-1*	209.09 (174–248)	217 (169–241)	215.50 (180–243)	—
* Weight-2*	259.81 (218–288)	310.63 (268–390)	305.36 (251–332)	—
* Weight-3*	271.54 (190–310)	333.63 (270–401)	331.58 (309–354)	275.9 (225–328)

The data are presented as Median (Range) (Groups: Control; Ovariectomy; Anastrazole; Letrozole).

**Table 2:** Serum glucose (mg/dl), total cholesterol (mg/dl), LDL-cholesterol (mg/dl), HDL-cholesterol (mg/dl) and triglycerides levels (mg/dl) at baseline (1), 2 months (2) and four months (3).

Variable	Median (range)Ovariectomy	Median (range) Anastrazole	Median (range) Letrozole	Median (range)Control group	p-value
Glucose and Lipid profile					
Glucose 1	155 (133–208)	143 (128–190)	139.5 (132–149)	—	.051
Glucose 2	144 (105–164)	150 (135–170)	145.5 (120–199)	—	.382
Glucose 3	139 (114–166)	141 (110–165)	158 (119–194)	156 (136–169)	.084
Cholesterol 1	54 (44–65)	54 (31–71)	55.5 (49–61)	—	.784
Cholesterol 2	48 (16–89)	60 (48–73)	72.5 (44–108)	—	.003
Cholesterol 3	49 (34–67)	55 (40–140)	60 (48–78)	51 (42–63)	.039
Triglycerides 1	66 (38–134)	69 (27–92)	65.5 (56–81)	—	.930
Triglycerides 2	50 (38–127)	135 (54–169)	81.5 (47–155)	—	.002
Triglycerides 3	66 (30–155)	96 (60–190)	137.5 (63–195)	132 (81–186)	.006
HDL 1	11 (10–13)	11 (10–25	13 (11–16)	—	.005
HDL 2	12 (10–15)	12 (10–24)	28.5 (10–49)	—	.004
HDL 3	12 (10–16)	11 (10–15)	11.5 (10–18)	13 (12–16)	.119
LDL 1	28 (19–37)	30 (15–46)	29 (19–39)	—	.749
LDL 2	24 (8–62)	22 (10–37)	32 (9–50)	—	.146
LDL 3	23 (5–66)	24 (12–91)	25 (8–63)	14 (1–30)	.038

The data are presented as Median (Range) (Groups: Control; Ovariectomy; Anastrazole; Letrozole). (Statistical significance was set at p < 0.5).

**Table 3:** Results of the liver pathology analysis.

Variable	Ovariectomy	Anastrazole	Letrozole	Controlgroup	p-value
Steatosis (0)	10/11	3/11	1/12	9/10	<.001
Steatosis (1)	1/11	8/11	9/12	1/10	
Steatosis (2)	0/11	0/10	2/12	0/11	
Steatosis location	0/11	0/10	2/12	0/11	.234
Ballooning (0)	11/11	9/11	5/12	10/10	.004
Ballooning (1)	0/11	2/11	5/12	0/10	
Ballooning (2)	0/11	0/11	2/12	0/10	
Total	1/11	8/11	11/12	1/10	<.001

No pathology (1), mild pathology (2), moderate pathology (3) (Groups: Control; Ovariectomy; Anastrazole; Letrozole).

## References

[b1] Yoshimoto F. K. & Guengerich F. P. Mechanism of the third oxidative step in the conversion of androgens to estrogens by cytochrome P450 19A1 steroid aromatase. Journal of the American Chemical Society 136, 15016–15025, 10.1021/ja508185d (2014). 25252141 PMC4210144

[b2] Stubert J., Dieterich M. & Gerber B. Medical prevention of breast cancer. Breast care 9, 391–396, 10.1159/000369573 (2014). 25759621 PMC4317676

[b3] Egbuta C., Lo J. & Ghosh D. Mechanism of inhibition of estrogen biosynthesis by azole fungicides. Endocrinology 155, 4622–4628, 10.1210/en.2014-1561 (2014). 25243857 PMC4239419

[b4] Elliott K. M. *et al.* Effects of aromatase inhibitors and body mass index on steroid hormone levels in women with early and advanced breast cancer. The British journal of surgery 101, 939–948, 10.1002/bjs.9477 (2014). 24687409

[b5] Koukouras D. *et al.* Association of estrogen receptor alpha (ERalpha) gene polymorphisms with endometrial thickness and lipid profile in women with breast cancer treated with aromatase inhibitors. Gynecological endocrinology: the official journal of the International Society of Gynecological Endocrinology 28, 859–862, 10.3109/09513590.2012.671393 (2012). 22799738

[b6] Lumachi F. *et al.* Endocrine therapy of breast cancer. Current medicinal chemistry 18, 513–522 (2011). 21143113 10.2174/092986711794480177

[b7] Inno A. *et al.* Anastrozole-related acute hepatitis with autoimmune features: a case report. BMC gastroenterology 11, 32, 10.1186/1471-230X-11-32 (2011). 21453541 PMC3076249

[b8] Aydin M. *et al.* Letrozole induces hepatotoxicity without causing oxidative stress: the protective effect of melatonin. Gynecological endocrinology: the official journal of the International Society of Gynecological Endocrinology 27, 209–215, 10.3109/09513590.2010.488769 (2011). 20528203

[b9] Tanii H., Shitara Y. & Horie T. Population pharmacokinetic analysis of letrozole in Japanese postmenopausal women. European journal of clinical pharmacology 67, 1017–1025, 10.1007/s00228-011-1042-3 (2011). 21494765

[b10] Hozumi Y. *et al.* The effect of exemestane, anastrozole, and tamoxifen on lipid profiles in Japanese postmenopausal early breast cancer patients: final results of National Surgical Adjuvant Study BC 04, the TEAM Japan sub-study. Annals of oncology: official journal of the European Society for Medical Oncology/ESMO 22, 1777–1782, 10.1093/annonc/mdq707 (2011). 21285133

[b11] Arimidex T. A. o. i. C. T. G. *et al.* Comprehensive side-effect profile of anastrozole and tamoxifen as adjuvant treatment for early-stage breast cancer: long-term safety analysis of the ATAC trial. The Lancet. Oncology 7, 633–643, 10.1016/S1470-2045(06)70767-7 (2006). 16887480

[b12] Breast International Group 1–98 Collaborative, G. *et al.* A comparison of letrozole and tamoxifen in postmenopausal women with early breast cancer. The New England journal of medicine 353, 2747–2757, 10.1056/NEJMoa052258 (2005). 16382061

[b13] Moro L. *et al.* Aromatase deficiency inhibits the permeability transition in mouse liver mitochondria. Endocrinology 151, 1643–1652, 10.1210/en.2009-1450 (2010). 20194728

[b14] Hewitt K. N., Pratis K., Jones M. E. & Simpson E. R. Estrogen replacement reverses the hepatic steatosis phenotype in the male aromatase knockout mouse. Endocrinology 145, 1842–1848, 10.1210/en.2003-1369 (2004). 14684602

[b15] Burstein H. J. *et al.* American Society of Clinical Oncology clinical practice guideline: update on adjuvant endocrine therapy for women with hormone receptor-positive breast cancer. Journal of clinical oncology: official journal of the American Society of Clinical Oncology 28, 3784–3796, 10.1200/JCO.2009.26.3756 (2010). 20625130 PMC5569672

[b16] Mouridsen H. *et al.* Superior efficacy of letrozole versus tamoxifen as first-line therapy for postmenopausal women with advanced breast cancer: results of a phase III study of the International Letrozole Breast Cancer Group. Journal of clinical oncology: official journal of the American Society of Clinical Oncology 19, 2596–2606 (2001). 11352951 10.1200/JCO.2001.19.10.2596

[b17] Patel S. A., Winkel M., Ali M. K., Narayan K. M. & Mehta N. K. Cardiovascular Mortality Associated With 5 Leading Risk Factors: National and State Preventable Fractions Estimated From Survey Data. Annals of internal medicine, 10.7326/M14-1753 (2015). 26121190

[b18] Kwagyan J. *et al.* Obesity and Cardiovascular Diseases in a High-Risk Population: Evidence-Based Approach to CHD Risk Reduction. Ethnicity & disease 25, 208–213 (2015). 26118150 PMC4487367

[b19] Jacob S. & Ferrieres J. [Epidemiology of coronary disease and prevention of cardiovascular diseases]. Soins; la revue de reference infirmiere, 32–35 (2015). 26040138

[b20] Jensen J., Nilas L. & Christiansen C. Influence of menopause on serum lipids and lipoproteins. Maturitas 12, 321–331 (1990). 2124647 10.1016/0378-5122(90)90012-u

[b21] Bhavnani B. R. & Stanczyk F. Z. Pharmacology of conjugated equine estrogens: efficacy, safety and mechanism of action. The Journal of steroid biochemistry and molecular biology 142, 16–29, 10.1016/j.jsbmb.2013.10.011 (2014). 24176763

[b22] Anan K. *et al.* Effects of toremifene and anastrozole on serum lipids and bone metabolism in postmenopausal females with estrogen receptor-positive breast cancer: the results of a 2-year multicenter open randomized study. Breast cancer research and treatment 128, 775–781, 10.1007/s10549-011-1608-x (2011). 21638048

[b23] Banerjee S. *et al.* Comparative effects of anastrozole, tamoxifen alone and in combination on plasma lipids and bone-derived resorption during neoadjuvant therapy in the impact trial. Annals of oncology: official journal of the European Society for Medical Oncology/ESMO 16, 1632–1638, 10.1093/annonc/mdi322 (2005). 16030027

[b24] Amir E., Seruga B., Niraula S., Carlsson L. & Ocana A. Toxicity of adjuvant endocrine therapy in postmenopausal breast cancer patients: a systematic review and meta-analysis. Journal of the National Cancer Institute 103, 1299–1309, 10.1093/jnci/djr242 (2011). 21743022

[b25] Colleoni M. *et al.* Analyses adjusting for selective crossover show improved overall survival with adjuvant letrozole compared with tamoxifen in the BIG 1-98 study. Journal of clinical oncology: official journal of the American Society of Clinical Oncology 29, 1117–1124, 10.1200/JCO.2010.31.6455 (2011). 21321298 PMC3083867

[b26] Strasser-Weippl K., Badovinac-Crnjevic T., Fan L. & Goss P. E. Extended adjuvant endocrine therapy in hormone-receptor positive breast cancer. Breast 22 Suppl 2, S171–175, 10.1016/j.breast.2013.07.033 (2013). 24074782

[b27] Arimidex T. A. O. I. C. T. G. *et al.* Effect of anastrozole and tamoxifen as adjuvant treatment for early-stage breast cancer: 100-month analysis of the ATAC trial. The Lancet. Oncology 9, 45–53, 10.1016/S1470-2045(07)70385-6 (2008). 18083636

[b28] Van Poznak C., Makris A., Clack G., Barlow D. H. & Eastell R. Lipid profiles within the SABRE trial of anastrozole with and without risedronate. Breast cancer research and treatment 134, 1141–1147, 10.1007/s10549-012-2147-9 (2012). 22763465 PMC3418141

[b29] Lin Y. *et al.* A prospective, randomized study on hepatotoxicity of anastrozole compared with tamoxifen in women with breast cancer. Cancer science 105, 1182–1188, 10.1111/cas.12474 (2014). 24975596 PMC4462391

[b30] Sawada S. *et al.* Effect of anastrozole and tamoxifen on lipid metabolism in Japanese postmenopausal women with early breast cancer. Acta oncologica (Stockholm, Sweden) 44, 134–141, 10.1080/02841860510007585 (2005). 15788292

[b31] Boccardo F. *et al.* Switching to anastrozole versus continued tamoxifen treatment of early breast cancer. Updated results of the Italian tamoxifen anastrozole (ITA) tria. Annals of oncology: official journal of the European Society for Medical Oncology/ESMO 17 Suppl 7, vii10–14, 10.1093/annonc/mdl941 (2006). 16760270

[b32] Elisaf M. S. *et al.* Effect of letrozole on the lipid profile in postmenopausal women with breast cancer. European journal of cancer 37, 1510–1513 (2001). 11506958 10.1016/s0959-8049(01)00155-1

[b33] Wasan K. M. *et al.* The influence of letrozole on serum lipid concentrations in postmenopausal women with primary breast cancer who have completed 5 years of adjuvant tamoxifen (NCIC CTG MA.17L). Annals of oncology: official journal of the European Society for Medical Oncology/ESMO 16, 707–715, 10.1093/annonc/mdi158 (2005). 15817595

[b34] Wasan K. M. *et al.* Lipid concentrations in postmenopausal women on letrozole after 5 years of tamoxifen: an NCIC CTG MA.17 sub-study. Breast cancer research and treatment 136, 769–776, 10.1007/s10549-012-2294-z (2012). 23089983

[b35] Yatsuji S., Hashimoto E., Tobari M., Tokushige K. & Shiratori K. Influence of age and gender in Japanese patients with non-alcoholic steatohepatitis. Hepatology Research 37, 1034–1043, 10.1111/j.1872-034X.2007.00156.x (2007). 17610504

[b36] Lemieux C. *et al.* Estrogen receptor [alpha]-mediated adiposity-lowering and hypocholesterolemic actions of the selective estrogen receptor modulator acolbifene. Int J Obes Relat Metab Disord 29, 1236–1244 (2005). 10.1038/sj.ijo.0803014 15925950

[b37] Yasutake K. *et al.* Nutritional investigation of non-obese patients with non-alcoholic fatty liver disease: The significance of dietary cholesterol. Scandinavian Journal of Gastroenterology 44, 471–477, 10.1080/00365520802588133 (2009). 19058085

[b38] Plourde P. V. *et al.* ARIMIDEX: a new oral, once-a-day aromatase inhibitor. The Journal of steroid biochemistry and molecular biology 53, 175–179 (1995). 7626450 10.1016/0960-0760(95)00045-2

[b39] Korou L. M. *et al.* Impact of N-acetylcysteine and sesame oil on lipid metabolism and hypothalamic-pituitary-adrenal axis homeostasis in middle-aged hypercholesterolemic mice. Scientific reports 4, 6806, 10.1038/srep06806 (2014). 25348324 PMC4210865

[b40] Kleiner D. E. *et al.* Design and validation of a histological scoring system for nonalcoholic fatty liver disease. Hepatology (Baltimore, Md.) 41, 1313–1321, 10.1002/hep.20701 (2005). 15915461

